# Taxonomical, Physiological, and Biochemical Characteristics of *Dunaliella salina* DSTA20 from Hypersaline Environments of Taean Salt Pond, Republic of Korea

**DOI:** 10.3390/microorganisms12122467

**Published:** 2024-11-30

**Authors:** Chang Rak Jo, Kichul Cho, Sung Min An, Jeong-Mi Do, Ji Won Hong, Ju Hyoung Kim, Sun Young Kim, Hyeon Gyeong Jeong, Nam Seon Kang

**Affiliations:** 1 National Marine Biodiversity Institute of Korea, Seocheon 33662, Republic of Korea; happyccr@mabik.re.kr (C.R.J.); kichul.cho@mabik.re.kr (K.C.); sman@mabik.re.kr (S.M.A.); sykim@mabik.re.kr (S.Y.K.); hgjeong@mabik.re.kr (H.G.J.); 2 Department of Biology, College of Natural Sciences, Kyungpook National University, Daegu 41566, Republic of Korea; jmdoe09@knu.ac.kr; 3 Department of Hydrogen and Renewable Energy, Kyungpook National University, Daegu 41566, Republic of Korea; jwhong@knu.ac.kr; 4 Advanced Bio-Resource Research Center, Kyungpook National University, Daegu 41566, Republic of Korea; 5 Department of Aquaculture and Aquatic Science, Kunsan National University, Gunsan 54150, Republic of Korea; juhyoung@kunsan.ac.kr

**Keywords:** *Dunaliella salina*, halophilic microalga, taxonomy, biodiesel production, fatty acid profile, carotenoids, monosaccharides, molecular identification

## Abstract

*Dunaliella salina*, a halophilic unicellular chlorophyte, produces bioactive compounds and biofuels applicable to various industries. Despite its industrial significance, comprehensive studies on the morphological, physiological, and biochemical characteristics of the genus *Dunaliella* remain challenging. In this study, we characterized an axenically isolated green alga from a salt pond in Taean, Republic of Korea, and assessed its industrially relevant traits. The morphological characteristics were typical of *D. salina*, and molecular phylogenetic analysis of the SSU, ITS1-5.8S-ITS, LSU regions of rDNA, and *rbc*L gene confirmed the isolate as *D. salina* strain DSTA20. The optimal temperature, salinity, and photon flux density required for its growth were determined to be 21 °C, 0.5 M NaCl, and 88 µmol m^−2^ s^−1^, respectively. Dried biomass analysis revealed 42.87% total lipids, with major fatty acids, including α-linolenic acid (31.55%) and palmitic acid (21.06%). The alga produced high-value carotenoids, including β-carotene (2.47 mg g^−1^ dry weight (DW)) and lutein (1.39 mg g^−1^ DW), with peak levels at 0.25 M salinity. Glucose (195.5 mg g^−1^ DW) was the predominant monosaccharide. These findings highlight the potential of *D. salina* DSTA20 for biodiesel production and as a source of ω-3 fatty acids, carotenoids, and glucose. Morphological traits provide insights relevant to the industrial potential of the species.

## 1. Introduction

Microalgae are remarkable in their ability to thrive under a variety of environments, including oceans, rivers, lakes, wetlands, and extreme conditions, such as salt flats, wastewater, hot springs, and the Antarctic region [1,2,3]. Notably, certain species are adapted to hypersaline environments with salt concentrations substantially higher than those of typical seawater (approximately 3.5% *w*/*v*), and even reaching up to 35% *w*/*v* [4]. These ecosystems harbor microorganisms with unique adaptations, offering both biodiversity and biotechnological potential [5].

The genus *Dunaliella* is distinguished among microalgae because of its remarkable capacity to thrive and survive in extreme environments [3]. As of date, 27 *Dunaliella* species have been identified, with 23 species found in marine and hypersaline environments and 4 in freshwater or brackish waters [6]. The *Dunaliella* species, especially the halophilic strains, produce considerable amounts of β-carotene, an antioxidant with significant commercial value [7]. These species thrive at salinities of 6–12% and regulate osmotic pressure by accumulating glycerol, which promotes the production of valuable compounds, such as lipids and carotenoids. These compounds are used in biofuels, cosmetics, and food additives [8,9].

Despite possessing several beneficial properties, *Dunaliella* presents challenges for industrial use, particularly with regard to species classification. Its morphological variability, which is influenced by environmental factors such as salinity, complicates its accurate identification [10]. Misidentification can have serious consequences, particularly when toxic microalgae species are involved [11]. In biotechnology, accurate taxonomy is essential for gene discovery, metabolic pathway studies, and the identification of valuable biological resources. The variability in physiological traits among strains, even within the same species, can affect metabolite production [12,13,14].

Among the various *Dunaliella* species, *Dunaliella salina* (Dunal) Teodoresco 1905 has emerged as one of the best microalgae for β-carotene production, making it a favorite among the most commercially important species. *D*. *salina* exhibits considerable industrial potential, particularly as an approved ingredient in functional foods, general foods, and cosmetics [15,16,17]. The global market for products related to *D*. *salina* was valued at USD 88 million in 2022, with an estimated compound annual growth rate of 4.4% from 2023 to 2032, driven by the demand for aquaculture and animal feed [18]. However, the industrial application of indigenous Korean strains remains underdeveloped, despite the likelihood that these strains possess unique traits suited to local environments. To realize their full potential, comprehensive studies on their taxonomic, genetic, physiological, and biochemical characteristics are necessary.

This study was aimed at exploring the taxonomic, physiological, and biochemical characteristics of the indigenous *D*. *salina* strain DSTA20 isolated from a salt pond in Taean-gun, Chungcheongnam-do, Republic of Korea. The specific objectives were to (1) identify the taxonomic, morphological, and molecular traits of the strain; (2) determine its optimal growth conditions under varying salinity, temperature, and light intensities; and (3) analyze its biochemical attributes, including fatty acid composition, carotenoid profile, and monosaccharide content. These findings should support the industrial application of *D*. *salina* in the Republic of Korea and expand its use in various industries.

## 2. Materials and Methods

### 2.1. Sample Collection and Isolation

**Figure 1:**
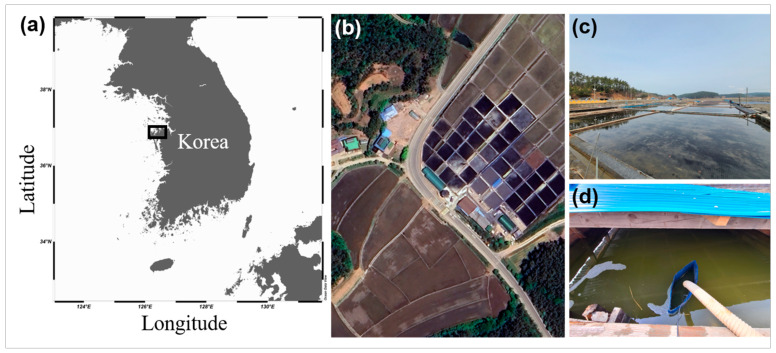
Location and photographs of the sampling site in the Naeri Mandae Solhyanggi Salt Pond, Republic of Korea. (**a**) Map of the sampling site: location of the site in the Naeri Mandae Solhyanggi Salt Pond on the west coast of Taean-gun in Chungcheongnam-do. (**b**) Environment surrounding the Naeri Mandae Solhyanggi Salt Pond in the Republic of Korea: an image acquired using Google Earth. (**c**) Overview of the site from which *Dunaliella salina* DSTA20 was collected. (**d**) Close-up of the specific locations from which *D. salina* DSTA20 samples were collected.

Plankton samples were collected from Naeri Mandae Solhyanggi Salt Pond (36°57′47.45″ N, 126°17′25.37″ E) in Taean-gun, Chungcheongnam-do, Republic of Korea, in July 2020. At the time of sampling, the temperature was recorded at 27.2 °C and the salinity of the pore water was greater than 100 practical salinity unit (PSU) (Table 1 and Figure 1).

The samples were gently filtered through a 154 μm Nitex mesh and placed into six-well tissue culture plates. Pure cultures of *D. salina* were established via two consecutive single-cell isolation steps using the Micropipette Washing Technique, as described in previous studies [19]. Isolated *D*. *salina* cells were immediately transferred to polycarbonate (PC) bottles containing f/2 medium (AusAqua, Wallaroo, South Australia, Australia), with the salinity adjusted to 0.5 M using salt collected from the salt pond (approximately 30 PSU). The bottles were sealed and incubated at 20 °C under cool white fluorescent lights, providing approximately 20 μmol photons m^−2^ s^−1^ in a 14:10 h light–dark cycle. As the density of *D*. *salina* increased, the cells were progressively transferred to larger PC bottles (50, 125, and 500 mL) containing fresh f/2 medium. Dense cultures were maintained by transferring the cells to fresh 500 mL PC bottles every four weeks.

### 2.2. Morphological Identification

The morphology of living cells grown photosynthetically was examined using an inverted microscope (CKX53; Olympus, Tokyo, Japan). The length and width of the live cells were measured using a digital camera (Zeiss AxioCam MRc5; Carl Zeiss, Göttingen, Germany).

For field emission scanning electron microscopy (FE-SEM), 10 mL aliquots of cultures were fixed in osmium tetroxide (OsO_4_; Electron Microscopy Sciences, Hatfield, PA, USA) at a density of approximately 1000 cells mL^−1^ and a final concentration of 1% (*v*/*v*) for 10 min. The fixed cells were collected on polycarbonate membrane filters with 3 µm pores (Whatman Nuclepore Track-Etched Membranes; Whatman, Kent, UK) and washed three times with distilled water. The membranes with attached cells were dehydrated in a graded ethanol series (10, 30, 50, 70, 90, and 100% ethanol), followed by two changes in 100% ethanol (Merck, Darmstadt, Germany). Subsequently, the samples were rapidly dried using an automated critical point dryer (EM CPD300, Leica, Wetzlar, Germany) with CO_2_. The dried filters were mounted on an aluminum stub (Electron Microscopy Sciences) using copper conductive double-sided tape (Ted Pella, Redding, CA, USA) and coated with gold using an ion sputter (MC1000, Hitachi, Tokyo, Japan). The cells and their surface morphologies were observed using a high-resolution Zeiss Sigma 500 VP FE-SEM (Carl Zeiss).

The cells were transferred to a 10 mL tube and fixed in 2.5% (*v*/*v*) glutaraldehyde (final concentration) for 1.5 h for transmission electron microscopy (TEM). The tube contents were placed in a 10 mL centrifuge tube and concentrated at 1610× *g* for 10 min in a centrifuge (VS-5500; Vision, Bucheon, Republic of Korea). The resulting pellet was transferred to a 1.5 mL tube and rinsed several times with 0.2 M sodium cacodylate buffer (pH 7.4) (Electron Microscopy Sciences). The cells were post-fixed with 1% (*w*/*v*) OsO_4_ in deionized water for 90 min. The pellet was embedded in agar before being dehydrated in a graded ethanol series (50, 60, 70, 80, 90, and 100% ethanol), followed by two changes in 100% ethanol. The material was then embedded in Spurr’s resin (Electron Microscopy Sciences). Sections were prepared using an EM UC7 ultramicrotome (Leica) and stained with 3% (*w*/*v*) aqueous uranyl acetate (Electron Microscopy Sciences) followed by 0.5% (*w*/*v*) lead citrate (Electron Microscopy Sciences). The sections were visualized using TEM (Sigma 500/VP TEM; Carl Zeiss).

### 2.3. Molecular Identification

Genomic DNA (gDNA) was extracted for molecular analysis using an AccuPrep Genomic DNA Extraction Kit (Bioneer, Daejeon, Republic of Korea), according to the manufacturer’s instructions. The primers used for amplifying each marker gene are listed in Table 2. The reaction mixtures for PCR amplification comprised 5 µL of 10× F-Star Taq Reaction Buffer, 1 µL of 10 mM dNTP mix, 0.02 µM of primers, 0.25 µL of 5 U/µL BioFACT F-Star Taq DNA polymerase (BioFACT Co., Ltd., Daejeon, Republic of Korea), 38.75 µL of UltraPure DNAse/RNAse-Free Distilled Water (Invitrogen, Carlsbad, CA, USA), and 3 µL of the DNA template (ca. 10–30 ng DNA). PCR amplification was performed on an Eppendorf Mastercycler PCR machine (Eppendorf, Hamburg, Germany) under the following thermal cycling conditions: pre-denaturation at 94 °C for 5 min, followed by 35 cycles of 94 °C for 1 min, annealing temperature (AT) for 1 min, 72 °C for 1 min, and a final extension at 72 °C for 5 min. The AT of the primers was determined using gradient PCR. The optimized ATs were as follows: 52.4 °C (EukA-G18R), 52.4 °C (570F-EukB), 52.4 °C (ITSF2-ITSFR2), 53.0 °C (D1R-LSUB), and 52.4 °C (*rbc*L-192- *rbc*L-657). The PCR products were purified using the AccuPrep PCR Purification Kit (Bioneer) and subjected to Sanger sequencing (Macrogen, Daejeon, Republic of Korea). Nucleotide sequences were identified using the Basic Local Alignment Search Tool provided by the National Center for Biotechnology Information (NCBI). Alignments and phylogenetic and molecular evolutionary analyses of the obtained sequences were conducted using the Geneious Prime v.2024.0.7. software (Biomatters Ltd., Auckland, New Zealand). This analysis incorporated various assemblages and drew on data from other species available in the NCBI GenBank database. Bayesian analyses were performed using MrBayes v.3.2.7 [20,21], with the GTR + G + I model applied to analyze the data from each region, offering a comprehensive framework for assessing sequence evolution. Four independent Markov chain Monte Carlo runs were executed for all sequence regions following the procedures outlined by Kang et al. [22]. In addition, maximum likelihood (ML) analyses were conducted using raxmlGUI 2.0 [23], also applying the GTR + G + I substitution model. Two hundred independent free inferences were allowed, using the –# option to identify the optimal tree. Bootstrap values were calculated with 1000 replicates, employing the same GTR + G + I model.

### 2.4. Determination of Optimal Culture Conditions

Routine serial subculturing was performed on the f/2 medium to maintain a pure culture of *D*. *salina* DSTA20. Initially, NaCl concentrations were adjusted to 0, 0.1, 0.25, 0.5, 1.0, 1.5, and 2.0 M in f/2 medium. The cultures were incubated at 20 °C in a stationary incubator (EYELA, Bunkyo-ku, Tokyo, Japan) for 30 days at each concentration to acclimate the cells to their respective salinity levels. Following this acclimatization period, an optimal culture test was conducted at the laboratory scale. In this phase, NaCl concentrations were maintained at 0, 0.1, 0.25, 0.5, 1.0, 1.5, and 2.0 M in the f/2 medium. A daily growth assessment was conducted over a period of 21 days under controlled conditions of 20 °C and a 14:10 h light–dark cycle with cool white fluorescent light at approximately 20 μmol photons m^−2^ s^−1^, all within the same stationary incubator to determine the optimal salinity. Daily growth was analyzed by counting cells using a DHC-N01 hemocytometer (INCYTO, Cheonan, Republic of Korea). As the optimal concentration was identified to be 0.5 M, cultures grown at this concentration were used for further experiments. Subsequently, the optimal temperature and illumination analyses were conducted simultaneously using PhotoBiobox [29]. A 200 µL algal culture aliquot was added to 96-well black/clear bottom plates and covered with a well plate-sealing film. After incubation for 72 h in a PhotoBiobox controlled at 5–40 °C and 0–350 µmol m^−2^ s^−1^, the optical density (OD) was measured at 600 nm using a Synergy II microplate reader (Biotek, Winooski, VT, USA). The OD at 600 nm was assumed to be proportional to the biomass of the species, and based on this assumption, the specific growth rate (μ) was calculated using the formula μ = (ln A_2_ − ln A_1_)/(T_2_ − T_1_), where A_1_ and A_2_ represent the O.D. values at T = 0 and T = 72 h, respectively. The calculated growth rates were visualized as heat maps using Microsoft Excel 2019 (Microsoft, Redmond, WA, USA).

### 2.5. Determination of Total Lipid, Fatty Acid Composition, Biodiesel Properties

Total lipid extraction was conducted on cultured samples grown under the optimal concentration (0.5 M), as described in the preceding experiment, with minor modifications to the methodology described by Folch et al. [30]. The algal culture medium (1 L) was harvested by centrifugation at 5000× *g* for 10 min and freeze-dried for 24 h (Kansas City, MO, USA). Subsequently, a chloroform–methanol (2:1) solution was added to 1 g of the freeze-dried lyophilized algal biomass. The mixture was then subjected to ultrasonic extraction using an Ultrasonic Processor (STH-1500S, Jeio Tech, Daejeon, Republic of Korea) for 30 min and filtered through a PTFE syringe filter (0.2 μm-pore size, Whatman). After drying overnight, the remaining total lipid residue was weighed using a MCA125P-2S00-U microbalance (Sartorius, Gottingen, Germany). The total lipid productivity was calculated using the following equation [31]:

Lipid productivity (g L^−1^ d ^−1^) = CL (g L^−1^)/t (1)

where CL (g L^−1^) is the content of lipid at the end of the mass culture run and t is the duration time of the cultivation.

The transesterification of the extracted total lipids was carried out by adding methanol containing 0.5% sodium methoxide and 2.5% H_2_SO_4_ (*v*/*v*) as a catalyst at 80 °C for 5 min [32]. The fatty acid methyl ester (FAME) composition was analyzed using a 7890A gas chromatograph equipped with a 5975C mass selective detector (Agilent, Palo Alto, CA, USA), as described in our previous publication [13]. Biodiesel properties, including the saponification value (SV), iodine value (IV), degree of unsaturation (DU), monounsaturated fatty acid (MUFA), polyunsaturated fatty acid (PUFA), long-chain saturation factor (LCSF), cold filter plugging point (CFPP), cetane number (CN), and oxidative stability (OS), based on the FAME profiles, were calculated in accordance with the method described by Islam et al. [33].

### 2.6. Microalgal Carotenoid Extraction and Analysis

Microalgal cells were acclimated for over 30 days to each NaCl concentration (0, 0.1, 0.25, 0.5, 1.0, 1.5, and 2.0 M) in f/2 medium. After acclimation, the cells were further cultivated for an additional 21 days under the same salinity conditions to reach higher cell densities. The harvested biomass was freeze-dried using a FreeZone 4.5 freeze dryer (Labconco, Kansas City, MO, USA). The freeze-dried samples (10 mg) were weighed and resuspended in methanol (1.5 mL) for carotenoid extraction and analysis. The samples were sonicated using an ultrasonicator at a resonance frequency of 40 Hz in an ultrasonic bath (Bransonic CPX5800H-E; Branson, Danbury, CT, USA) for 90 min. The samples were then centrifuged at 16,022× *g* and 4 °C for 20 min, and the supernatant was collected. The supernatant was evaporated using a rotary evaporator (IKA RV; IKA, Staufen, Germany), and the dried sample was dissolved in acetone (1.5 mL) and filtered through a 0.2 μm membrane filter (Minisart syringe filter; Sartorius, Göttingen, Germany) for HPLC analysis. The HPLC analysis was conducted according to the method described by Yang et al. [34]. The carotenoids were then analyzed using an Agilent 1200 series gradient HPLC system (Agilent Technologies, Palo Alto, CA, USA) equipped with a C30 carotenoid column (250 mm × 4.6 mm, 5 μm, YMC, Kyoto, Japan). The solvent system comprised 92% methanol, 10 mM ammonium acetate (solvent A), and tert-butyl methyl ether (solvent B) and was passed through the column at a constant flow rate of 1 mL/min for 1 h. Carotenoid standards, including astaxanthin, β-carotene, canthaxanthin, lutein, and zeaxanthin, were purchased from Sigma-Aldrich (St. Louis, MO, USA). The profiles of the standards and the extracted pigments were determined by measuring their OD at 450 nm.

### 2.7. Extraction and Analysis of Microalgal Monosaccharides

Cultured samples grown under the optimal concentration (0.5 M) identified in the preceding experiment were further cultivated for an additional 21 days under the same salinity conditions to reach higher cell densities. For monosaccharide analysis, 50 mg of freeze-dried biomass was hydrolyzed in 2.5 mL of 2 N sulfuric acid at 94 °C for 3 h. After cooling to room temperature (20 °C) and neutralizing with CaCO_3_, the sample was filtered through a 0.2 μm PTFE filter (Whatman). The analysis was conducted using an Alliance HPLC system (Waters Co., Milford, MA, USA) equipped with a Sugar-Pak I column (Ø 6.5 × 300 mm, Waters Co.). The mobile phase was 0.01 M Ca-EDTA (50 mg L^−1^ distilled H_2_O), with a flow rate of 0.5 mL min^−1^ and a column temperature of 90 °C. A 20 μL sample was injected and analyzed using a refractive index detector. The total peak area of each monosaccharide was calibrated against standard substances (sucrose, lactose, glucose, galactose, fructose, arabinose, mannitol, and sorbitol; Sigma-Aldrich) to quantify the monosaccharide content (mg g^−1^) of the dry weight (DW) biomass.

### 2.8. Statistical Analysis

All experiments were performed in triplicate, and the data are expressed as the mean ± standard error and standard deviation. The total lipid content was analyzed using one-way analysis of variance (ANOVA), followed by Tukey’s honest significant difference (HSD) test, using SPSS v.14.0 software, accessed on 1 October 2024. (IBM, SPSS Inc., Armonk, NY, USA) to assess differences between means. The remaining data are shown as the mean of three replicates.

## 3. Results

### 3.1. Morphological Characteristics

**Figure 2:**
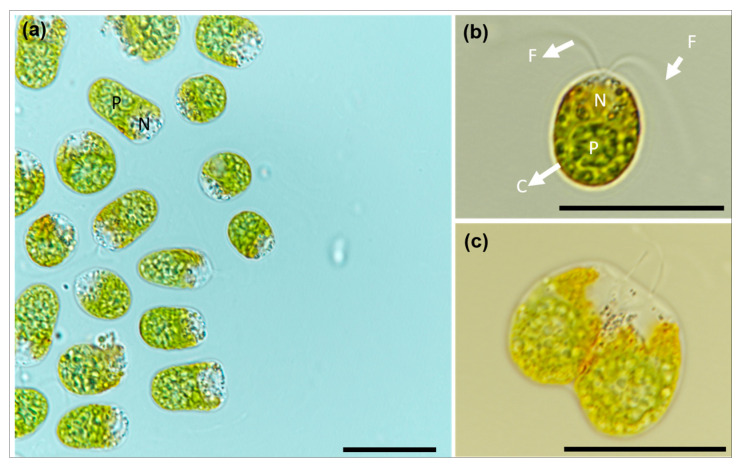
Light micrographs of *Dunaliella salina* DSTA20. (**a**) Vegetative cells with spheroidal to ellipsoidal shapes containing a nucleus (N) and a pyrenoid (P). (**b**) Single vegetative cell showing a cup-shaped chloroplast (C), two motile flagella (F), and a centrally positioned nucleus (N) with a pyrenoid (P). (**c**) Dividing cells are shown. Scale bars: (**a**–**c**) = 20 μm.

Single vegetative cells of *D. salina* DSTA20 in culture were nearly spherical to ellipsoidal in shape and appeared green to slightly orange (Figure 2a). The ranges (mean ± standard error, *n* = 30) of the cell lengths and widths were 9.3–14 μm (11.3 ± 1.1) and 7.8–10 μm (8.1 ± 0.8), respectively (Table 3). These cells exhibited two motile flagella that were approximately equal to or slightly longer than the cell length, inserted at the anterior end of the cell (Figure 2b). A cup-shaped chloroplast was observed along the cell periphery, with a single pyrenoid visible in vegetative cells (Figure 2b). A single nucleus was located adjacent to the pyrenoid (Figure 2b), and cells undergoing division were observed (Figure 2c).

**Figure 3:**
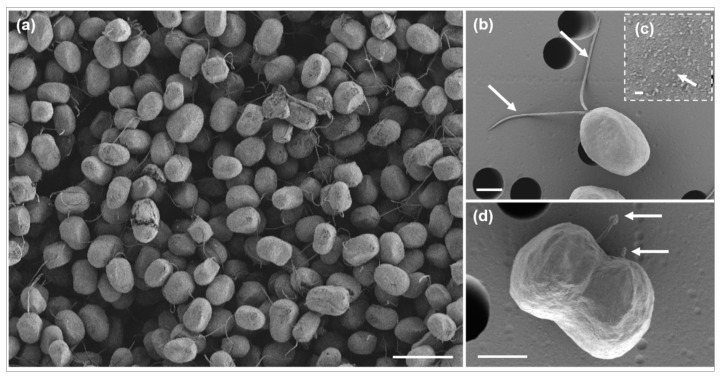
Scanning electron micrographs of *Dunaliella salina* DSTA20. (**a**) Group of cells displaying diverse forms and sizes, ranging from globose to ellipsoidal. (**b**) Single cell with two motile flagella (arrows). (**c**) A detailed view of the cell surface with small visible granules (arrow). (**d**) Dividing cells are shown, with the flagella visible. Scale bars: (**a**) = 20 μm, (**d**) = 4 μm, (**b**) = 3 μm, (**c**) = 0.2 μm.

Scanning electron micrographs revealed various forms of vegetative and reproductive cells in *D*. *salina* DSTA20 (Figure 3). The cells exhibited a range of shapes from globose to ellipsoidal, with an almost smooth surface structure, and they varied in size (Figure 3a). Each cell possessed two flagella that were slightly longer than or equal in length to the cell body (Figure 3b). In addition, cells undergoing division in the presence of flagella were observed (Figure 3d).

**Figure 4:**
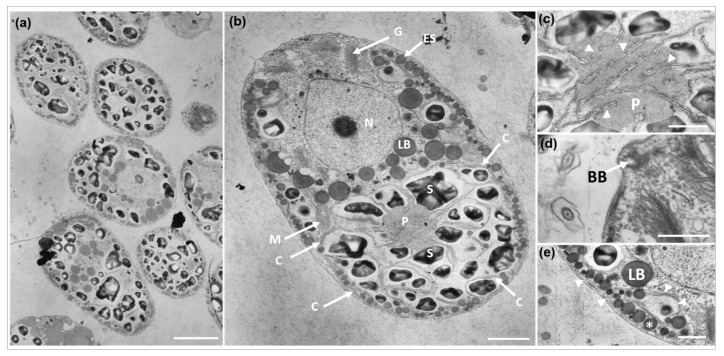
Transmission electron micrographs of *Dunaliella salina* DSTA20. (**a**) Various shapes and sizes of *D. salina* DSTA20 cells. (**b**) A micrograph showing the chloroplast (C), Golgi apparatus (G), eyespot (ES), lipid body (LB), mitochondria (M), nucleus (N), pyrenoid (P), and starch granules (S). (**c**) Detailed view of the pyrenoid (P) with thylakoid penetration. Small arrows indicate thylakoids. (**d**) Detailed view of the flagellar basal body (BB). (**e**) The eyespot (*) was located within the cup-shaped region of the chloroplast, with small arrows indicating the chloroplast structure. Lipid bodies (LBs) are also visible. Scale bars: (**a**) = 5 μm, (**b**) = 2 μm, (**c**–**e**) = 1 μm.

Transmission electron micrographs revealed the various shapes, sizes, and main ultrastructures of *D. salina* DSTA20 (Figure 4). The thin sections prepared for TEM clearly showed the primary cellular features, including the chloroplast (C), eyespot (ES), Golgi apparatus (G), lipid body (LB), mitochondria (M), nucleus (N), pyrenoid (P), and starch granules (S) (Figure 4b). Chloroplasts were observed along the periphery of the cells, with a single pyrenoid located centrally, surrounded by starch grains, and penetrated by thylakoids into the pyrenoid matrix (Figure 4b,c, Table 3). Numerous lipid bodies (LB) were also prominent within the cells (Figure 4b,e). The flagellar basal body (BB) was distinctly visible at the anterior end of the cell (Figure 4d), and an eyespot was observed within the cup-shaped region of the chloroplast (Figure 4e).

**Figure 5:**
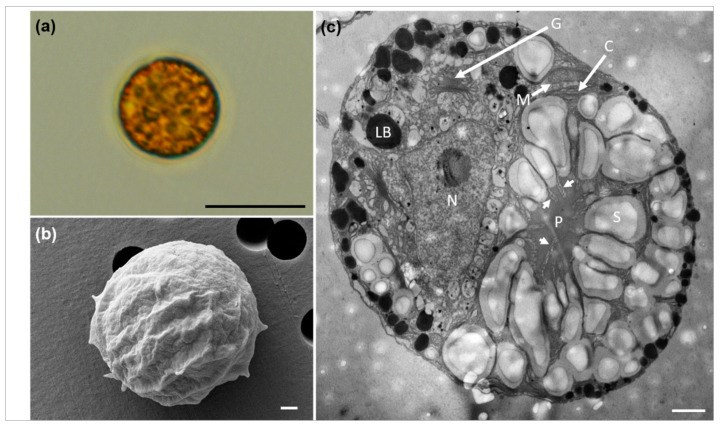
Light, scanning electron, and transmission electron micrographs of *Dunaliella salina* DSTA20 cultivated under low-salinity conditions (0.25 M NaCl). (**a**) Light micrograph showing aplanospore morphology. (**b**) Scanning electron micrograph of a *D. salina* cell, highlighting its rough surface. (**c**) Transmission electron micrograph showing the chloroplasts (C), Golgi apparatus (G), lipid body (LB), mitochondria (M), nucleus (N), pyrenoids (P), and starch granules (S). Arrows indicate the thylakoid membranes that penetrate the pyrenoid matrix. Scale bars: (**a**) = 10 μm, (**b**,**c**) = 1 μm.

Figure 5 presents the light, scanning electron, and transmission electron micrographs of *D. salina* DSTA20 cultivated under 0.25 M NaCl conditions. The cells exhibited an aplanospore morphology without visible flagella (Figure 5a). The cell diameter (mean ± standard error, *n* = 10) ranged from 12–19 μm (14.6 ± 0.8) (Table 3).

In the SEM image (Figure 5b), the cells appeared nearly spherical with a rough surface texture. TEM (Figure 5c) revealed that most cells were spherical in shape, with the main ultrastructures including the chloroplast (C), Golgi apparatus (G), lipid body (LB), mitochondria (M), nucleus (N), pyrenoid (P), and starch granules (S). A large pyrenoid (P), surrounded by starch grains, was observed in the center. In addition, thylakoid penetration into the pyrenoid matrix was observed, as indicated by arrows (Figure 5c).

### 3.2. Molecular Identification and Sequence Analysis

The total length of the sequences from the small subunit (SSU, 18S) rDNA, including the ITS1-5.8S rRNA-ITS2 regions, the large subunit (LSU, 28S) rDNA, and the *rbc*L gene, which is part of the chloroplast genome and encodes the large subunit of ribulose-1,5-bisphosphate carboxylase/oxygenase (RuBisCO), in the newly isolated strain, was 3153 nucleotides (GenBank accession numbers: PP973483, PP974559, PP973494, and PP975467; Table 1). The alignment results revealed that the SSU rDNA sequence of the *D. salina* DSTA20 isolate matched exactly with those of the *D. salina* strains CCAP 19/18 (Australia), SAG 42.88 (Israel), CCAP 19/12 (Israel), and KMMCC 1428 (Republic of Korea), as well as with an unidentified strain GY-H13. However, strains UTEX LB 1644 (USA) and KU07 (Thailand) exhibited variations with two to seven base substitutions in SSU compared with DSTA20 (Table 4).

**Figure 6:**
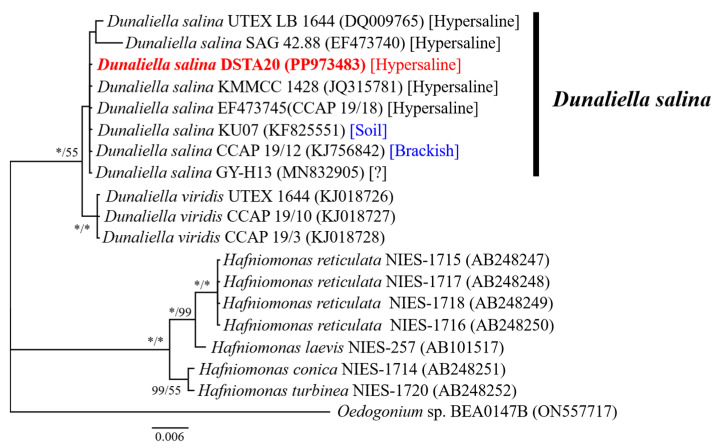
Maximum likelihood and Bayesian inference phylogenetic tree based on 18S rDNA sequences. The values on each node indicate maximum likelihood bootstrap and Bayesian posterior probabilities (%). The bootstrap values < 50 and Bayesian posterior probabilities < 75 are omitted. Red represents the strains we analyzed, blue represents non-hypersaline strains, and the question mark represents strains with unknown habitats. The scale bar shows nucleotide changes per site. * indicates 100.

**Figure 7:**
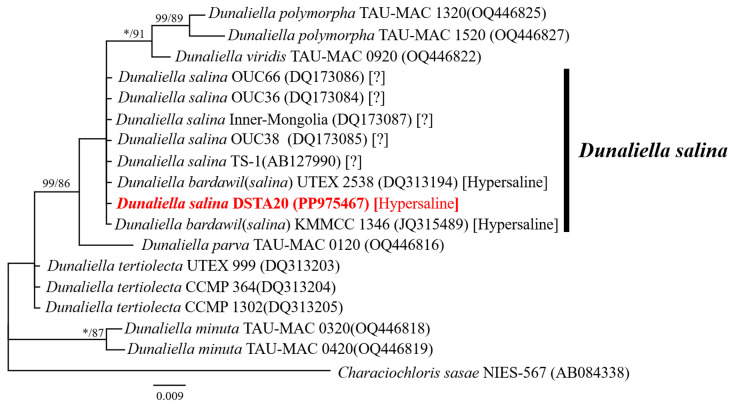
Maximum likelihood and Bayesian inference phylogenetic tree based on the *rbc*L sequences. The values on each node indicate maximum likelihood bootstrap and Bayesian posterior probabilities (%). The bootstrap values < 50 and Bayesian posterior probabilities < 75 are omitted. Red represents the strains we analyzed, and the question mark represents strains with unknown habitats. The scale bar shows nucleotide changes per site. * indicates 100.

In the phylogenetic tree based on SSU rDNA sequences, *D. salina* DSTA20 was grouped with other *D. salina* strains, including UTEX LB 1644, SAG 42.88, KMMCC 1428, CCAP 19/18, KU07, CCAP 19/12, and GY-H13 (Table 4, Figure 6). Additionally, in the phylogenetic tree based on *rbc*L sequences, *D. salina* DSTA20 was grouped with other *D. salina* strains, including OUC66, OUC36, Inner Mongolia, OUC38, TS-1, UTEX2538, and KMMCC1346 (Figure 7). Molecular characterization based on sequence analyses of SSU rDNA (Table 4, Figure 6) and *rbc*L (Figure 7) confirmed that the isolate belonged to the *D. salina* group. Consequently, this microalga was identified as *D. salina* DSTA20 and deposited at the National Marine Biodiversity Institute of Korea (MABIK) and the Korean Collection for Type Cultures under the accession numbers MABIK LP00000338 and KCTC 15718BP, respectively.

### 3.3. Verification of the Optimal Cultivation Conditions of the Isolated Strain

To verify the optimal cultivation conditions for the isolated algal strain, growth responses to different salinities, temperatures, and light intensities were assessed under laboratory conditions.

**Figure 8:**
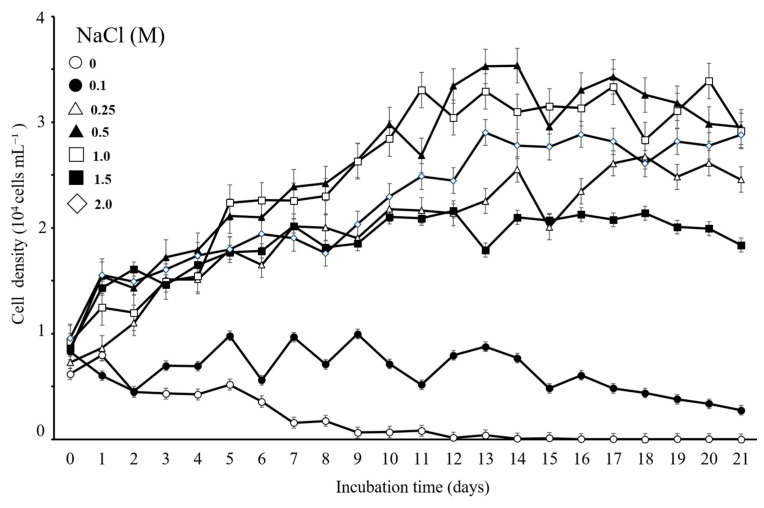
Effect of salt (NaCl, M) supplementation on the density of *Dunaliella salina* DSTA20. Data are presented as the mean values ± standard deviation (*n* = 3).

As shown in Figure 8, the effect of NaCl supplementation on the density of *D*. *salina* DSTA20 cells over the 21-day cultivation period indicated an increasing trend at salinity concentrations of 0.25, 0.5, 1.0, 1.5, and 2.0 M compared to the initial density, with optimal growth observed at 0.5 M NaCl. The growth phase began to accelerate within the first few days post-inoculation, with optimal growth observed at a 0.5 M NaCl concentration, where cell densities reached 2.4 × 10^4^ cells mL^−1^ by day 7. After 14 days, the cell density at 0.5 M NaCl further increased to 3.54 × 10^4^ cells mL^−1^, representing an approximately fivefold increase from the initial count. Additionally, the strain showed growth at the lowest concentration (0.1 M), whereas no growth was observed at 0 M NaCl.

**Figure 9:**
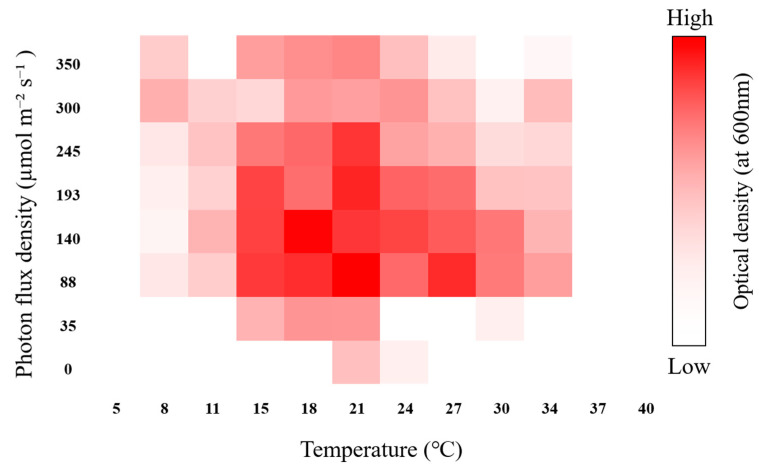
Heat map representing the screening of the algal growth (optical density at 600 nm) response, as determined via photobiobox analysis under different light intensities and temperatures.

As shown in Figure 9, *D. salina* DSTA20 grew at a temperature between 8 and 34 °C, and the highest growth rate was determined at 18–27 °C and 88–245 µmol photons m^−2^ s^−1^ of photon flux density (PFD). The optimal conditions were determined to be 21 °C and 88 µmol photons m^−2^ s^−1^.

### 3.4. Proximate Composition and FAME Analysis, Along with the Evaluation of Biodiesel Properties

**Figure 10:**
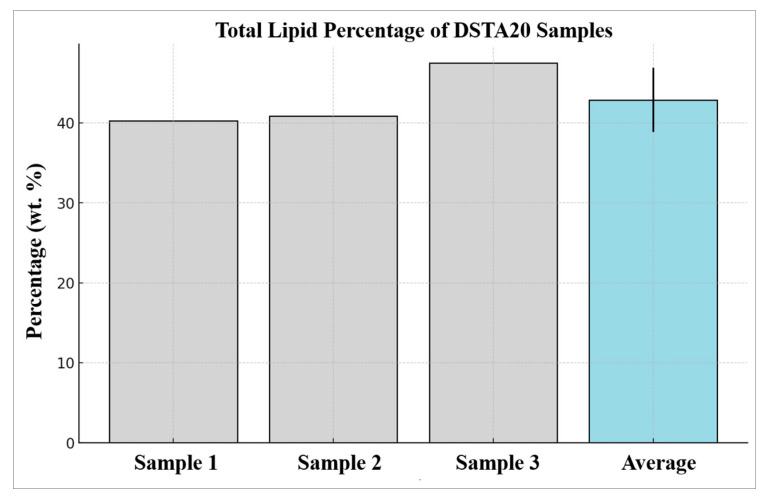
Total lipid content of *Dunaliella salina* DSTA20 cultivated in f/2 medium under photoautotrophic conditions. Error bars indicate the mean ± S.D., with significant differences indicated at *p* < 0.05.

The analysis of dried algal biomass under 0.5 M NaCl conditions, chosen considering the highest observed growth rate in the optimal cultivation conditions study, revealed a significant accumulation of total lipid content. As shown in Figure 10, total lipid was the predominant component, averaging 42.87% (*w*/*w*) for the three samples, with individual lipid percentages ranging from 40.28% to 47.49%.

The major FAMEs identified in this strain were α-linolenic acid (C_18:3_ ω-3, 31.55%), palmitic acid (C_16:0_, 21.06%), hexadecatetraenoic acid (C_16:4_ ω-3, 13.23%), and linoleic acid (C_18:2_ ω-6, 6.81%) (Table 5). Additionally, trace amounts of saturated fatty acids (SFAs), such as myristic acid (C_14:0_, 0.49%) and stearic acid (C_18:0_, 0.53%), were detected, accounting for 22.08% of the fatty acid content (Table 5). In contrast, total monounsaturated (MUFAs) and polyunsaturated (PUFAs) fatty acids accounted for 4.40% and 59.26% of the total fatty acid content, respectively (Table 5).

*D. salina* DSTA20 exhibited an SV, IV, DU, MUFA content, PUFA content, LCSF, CFPP, CN, and OS values of 171.39, 169.05, 122.92, 4.4%, 59.26%, 2.37, −9.03, 35.04, and 8.7, respectively (Table 6).

### 3.5. Analysis of Microalgal Carotenoid Profile

**Figure 11:**
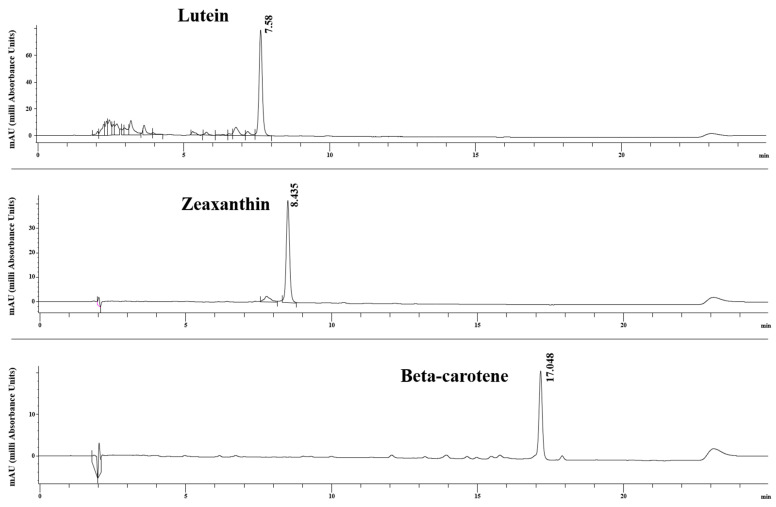
Chromatograms displaying the major carotenoids β-carotene, lutein, and zeaxanthin extracted at 0.25 M salinity.

The carotenoid profiles of *D. salina* DSTA20 are listed in Table 7 and illustrated in Figure 11. The carotenoid composition varied across different salinity conditions, with the highest concentrations recorded at 0.25 M. In particular, the amounts at 0.25 M were as follows: β-carotene, 2.47 mg g^−1^ DW; lutein, 1.39 mg g^−1^ DW; and zeaxanthin, 0.69 mg g^−1^ DW. At 0.1 M salinity, the detected concentrations were 0.98 mg g^−1^ DW for β-carotene and 0.73 mg g^−1^ DW for lutein; however, zeaxanthin was not detected. At higher salinity levels (0.5, 1, 1.5, and 2 M), the concentrations fluctuated. β-carotene was 1.27 mg g^−1^ DW at 0.5 M and 1.26 mg g^−1^ DW at 1 M. Lutein peaked at 1.06 mg g^−1^ DW at 1 M, with reduced concentrations observed at higher salinities, and zeaxanthin remained consistently low across all these conditions.

### 3.6. Analysis of the Monosaccharide Profile

The monosaccharide composition of *D. salina* DSTA20 is summarized in Table 8. Glucose was the predominant monosaccharide, with the DSTA20 strain exhibiting the highest concentration of 195.5 mg g^−1^ DW. Galactose was the next most abundant monosaccharide at 15.7 mg g^−1^ DW, followed by fructose and sucrose, which were present at 13.2 and 7.13 mg g^−1^ DW, respectively.

## 4. Discussion

Research on microalgae, particularly on the halophilic green unicellular flagellate *D. salina*, continues to be of great interest because of its exceptional ability to produce valuable bioactive compounds [44,45]. Despite extensive studies on *D. salina* for its β-carotene and glycerol production potential [9,46], ongoing research is crucial to further optimize its cultivation and enhance its industrial exploitation. In this study, we further investigated *D. salina*, specifically the strain DSTA20, to examine its taxonomic and physiological responses under varying conditions and to assess its bioactive compound production potential.

### 4.1. Morphological and Molecular Identification

*D*. *salina* DSTA20 exhibited unicellular, solitary cells without a rigid cell wall or lorica, and possessed two naked flagella, consistent with the characteristic features of the family Dunaliellaceae [47]. The observed morphological characteristics of *D*. *salina* DSTA20 closely aligned with those of the genus *Dunaliella*, as originally described by Teodoresco in 1905 [35]. Specifically, *D*. *salina* DSTA20 displayed an ellipsoid or ovoid cell shape, with parietal and primarily cup-shaped chloroplasts. The presence of a pyrenoid and a stigma (eyespot) further supports its classification within this genus. Additionally, the nucleus was consistently located at the anterior end, and the hematochrome pigment masked the cytoplasmic organization, in line with traits typical of *Dunaliella* members [35].

*D*. *salina* DSTA20 shares several key morphological and ultrastructural characteristics with other *D*. *salina* strains documented in previous studies, including those reported by Teodoresco [35], Borowitzka and Siva [10], and Highfield et al. [36]. These shared traits conclusively identify our isolate as *D. salina*. To confirm the taxonomic classification, we conducted molecular marker sequencing to support the morphological identification of *D. salina* DSTA20. The SSU rDNA sequence of *D. salina* DSTA20 was identical to that of several other strains, including CCAP 19/18, SAG 42.88, CCAP 19/12, KMMCC 1428, and GY-H13. Additionally, the *rbc*L chloroplast gene sequence of *D. salina* DSTA20 was identical to that of strains OUC66, OUC36, Inner Mongolia, OUC38, TS-1, UTEX2538, and KMMCC1346. Phylogenetic analysis further confirmed that the DSTA20 strain belongs to *D. salina*, based on both SSU and *rbc*L gene sequences. The taxonomy of *D. salina* remains ambiguous, prompting us to perform ITS region sequencing and construct a phylogenetic tree for a more precise classification [48]. Our phylogenetic analysis revealed that the DSTA20 strain belongs to *D. salina* clade 2, which is characterized by distinct genetic features within *D. salina* and clearly separates it from *D. viridis* and *D. tertiolecta* (Figure S1). In this phylogenetic tree, the major species within the genus *Dunaliella* form independent clades, with *D. salina* comprising multiple clades that reflect divergent evolutionary paths from *D. viridis* and *D. tertiolecta* (Figure S1). The placement of DSTA20 within *D. salina* clade 2 indicates a close relationship with other *D. salina* strains and suggests potential shared adaptations to specific environmental conditions. These results clarify the taxonomic position of the DSTA20 strain at the molecular level, providing important insights into the phylogenetic diversity and evolutionary relationships within the genus *Dunaliella*. This analysis reinforces DSTA20 identification as *D. salina* and further distinguishes it phylogenetically from other *Dunaliella* species. Consequently, the green alga was conclusively identified as *D. salina* through genetic analysis.

Observations of *D. salina* DSTA20 under low-salinity conditions (0.25 M NaCl) provided detailed insights into its morphology and internal structure. Light microscopy and SEM revealed that the cells adopted an aplanospore form, characterized by a nearly spherical shape with a rough surface texture, which is consistent with previous reports [49,50]. Notably, this study demonstrated that aplanospore formation can occur at salinity levels even lower than the previously reported 5–10% NaCl (0.85 to 1.71 M) range, suggesting a broader adaptive capacity for this species.

Previous studies have shown that the *Dunaliella* species, including *D. salina*, form vegetative cysts (aplanospores) in response to reduced salinity, cooler temperatures, short-day conditions, and nutrient depletion [49,51,52,53,54,55,56]. Our findings confirm that *D. salina* DSTA20 forms aplanospores in 0.25 M NaCl, highlighting its adaptability to low-salinity stress. The aplanospores observed in this study exhibited extremely resistant, thick, two-layered, and rugose walls, which are consistent with the descriptions by Leonardi and Cáceres [50].

The similarity with other *D*. *salina* strains, such as MUR8, MUR9, and MUR22 [10], supports the hypothesis that aplanospore formation is a crucial mechanism of survival. The ability to form aplanospores in response to fluctuating salinity levels underscores the remarkable resilience of *D*. *salina* DSTA20, reinforcing its adaptive strategy of maintaining cellular function under low-salinity stress.

### 4.2. Ecological and Growth Characteristics, Including Adaptability

*D. salina* is a hypersaline organism that thrives in both natural and artificial saltwater environments worldwide, from Antarctica to subequatorial desert regions [10]. It typically flourishes at salinity levels ranging from approximately 5% (approximately 50 PSU) to saturation, with an optimal range of 20–25% (200–250 PSU) NaCl [10].

Reflecting this adaptability, *D. salina* strains have been identified in saline environments of various countries, including Algeria, Australia, India, Iran, Republic of Korea, Mexico, Russia, and Spain [49,57,58,59,60,61,62,63]. Whereas most *D. salina* strains inhabit high-salinity environments, some strains, such as the CCAP 19/12 strain from Israel, have been found in brackish waters, and the KU07 strain has been isolated from soil. In this study, the DSTA20 strain was isolated from hypersaline water (above 100 PSU) in the Republic of Korea, consistent with its preference for high-salinity environments. However, the adaptability of this strain suggests that *D. salina* can inhabit diverse habitats.

Our growth experiments demonstrated that *D. salina* DSTA20 is capable of surviving and growing across a wide salinity range, from 0.25 to 2.0 M NaCl. Although the growth density decreased at 0.1 M NaCl, the cells were able to maintain their activity. These results indicate that *D. salina* DSTA20 is not strictly limited to hypersaline conditions but can adapt to various salinity levels, including brackish waters. This adaptability aligns with reports of *D. salina* strains thriving in diverse environments such as soil, brackish water, and hypersaline habitats. Overall, our findings highlight the ecological versatility of *D. salina* DSTA20 and confirm its potential for survival and growth under a broad spectrum of salinity conditions, which may have significant implications for its ecological role and potential industrial applications.

The growth experiment demonstrated that *D*. *salina* DSTA20 achieved optimal growth at 0.5 M NaCl, with significant growth observed across a wide salinity range from 0.25 to 2.0 M NaCl. This indicates that although 0.5 M NaCl is ideal for biomass accumulation, the strain can adapt to various salinity levels. Its ability to grow under fluctuating salinity suggests a high level of adaptability, which makes it suitable for large-scale outdoor cultivation in diverse aquatic environments. Therefore, *D*. *salina* DSTA20 has strong potential as a valuable resource for biotechnological applications.

The growth conditions of *D. salina* DSTA20 were significantly influenced by temperature and PFD, which are crucial factors for maximizing microalgal growth [64]. In this study, *D. salina* DSTA20 demonstrated optimal growth within the temperature range of 18–27 °C and PFD between 88 and 245 µmol m^−2^ s^−1^. In particular, the most favorable growth was observed at 21 °C and 88 µmol m^−2^ s^−1^, which aligns closely with the previously reported findings for another *D. salina* strain, PSBDU05, which exhibited high growth rates at 23 °C [64]. This highlighted the thermal flexibility of this strain and its ability to adapt to a wide range of environmental temperatures.

*D. salina* DSTA20 also exhibited adaptability across a broad range of PFD, which reinforces its potential to thrive under different light conditions. When combining this broad adaptability to both temperature and PFD, it becomes evident that *D. salina* DSTA20 possesses significant potential to thrive under diverse environmental conditions. This adaptability explains the ability of this strain to inhabit a wide range of ecosystems, making it a promising candidate for biotechnological applications.

### 4.3. Lipid Contents

The total lipid content of *D. salina* DSTA20 under 0.5 M NaCl conditions revealed substantial lipid accumulation, averaging 42.87% (*w*/*w*), which is notably higher than the typical lipid content observed in many green microalgal species under normal conditions. For comparison, microalgae, such as *Botryococcus braunii*, *Graesiella emersonii*, *Nannochloropsis oculata*, and *Tetradesmus obliquus*, typically exhibit lipid contents ranging from 13 to 31% under non-optimized conditions [65]. The relatively high lipid accumulation in *D. salina* DSTA20 indicates its potential as a feedstock for biodiesel production, as lipid productivity is a crucial factor in evaluating microalgae for biofuel applications [66]. As observed in other studies, optimizing cultivation conditions, such as nutrient limitation, salinity stress, and heterotrophic growth, can significantly enhance lipid accumulation in microalgae [67,68,69]. Therefore, future research should focus on further optimizing the growth conditions of *D. salina* DSTA20 to increase its lipid productivity, which would make it an even more viable option for biodiesel production on a larger scale.

### 4.4. Fatty Acid Composition and Biodiesel Properties

The fatty acid profile of *D. salina* DSTA20 under 0.5 M NaCl conditions revealed richness in α-linolenic acid (ALA) at 31.55%, palmitic acid at 21.06%, and hexadecatetraenoic acid at 13.23% (Table S1). Notably, the presence of ALA as the major component is particularly significant because this ω-3 fatty acid plays a crucial role in human health, including anti-inflammatory effects and cardiovascular protection, and is typically sourced from fish and plants [70]. The ALA content of *D. salina* DSTA20 (31.55%) was generally consistent with the trend observed among *D. salina* strains, which typically maintain an ALA level of approximately 30%. Although certain strains, such as *D. salina* ITC5.103 (35.95%) and *D. salina* Y6 (39.51%), exhibited higher ALA content, DSTA20 still surpassed other microalgal species, such as *Chlorella salina* (16.4%) and *Graesiella emersonii* (27.2%), in terms of ALA concentration (Table S1). This comparison highlights the potential of *D. salina* DSTA20 as a significant source of ALA. Furthermore, DSTA20 demonstrates strong promise as a valuable resource for industries focusing on biofuel production and nutraceutical applications.

Compared with second-generation oil sources, *D. salina* DSTA20 far surpasses crops such as Jatropha (0.2% ALA), which reinforces its candidature as a valuable alternative to ω-3 PUFA supplementation. Although plant-based products are widely available, they require a long time to harvest [71]. Moreover, fish oils, which are rich in ω-3 fatty acids, are associated with environmental concerns, such as overfishing and contamination from pollutants [72,73]. In contrast, microalgae are more sustainable and environmentally friendly alternatives [71]. The cultivation of microalgae requires substantially less land and water resources than conventional agricultural crops [74]. Among the diverse species of microalgae, *D*. *salina*, particularly strain DSTA20, has demonstrated considerable potential because of its elevated ALA content. This strain is a renewable and scalable source of ALA, making it a sustainable alternative to ω-3 supplements. By overcoming the limitations associated with fish and plant sources, *D*. *salina* DSTA20 offers nutritional and environmental advantages as an effective source of ω-3 polyunsaturated fatty acids (PUFAs).

The palmitic acid content (C_16:0_, 21.06%) in *D. salina* DSTA20 is particularly noteworthy because of its industrial relevance in food production and biodiesel synthesis [75,76]. The combination of palmitic acid and other SFAs, such as stearic acid (C_18:0_, 0.53%), resulted in a total SFA content of 22.08%, which contributed to the stability of the lipid and its CN, which is desirable for biodiesel fuels [77].

Compared with other microalgal strains, *D. salina* DSTA20 demonstrated competitive palmitic acid levels. *D. salina* ITC5.103 had a slightly higher content (23.43%), whereas *D. salina* Y6 (19.73%) and *D. salina* CCAP 19/12 (12.16%) had lower amounts. Other species, such as *Chlorella vulgaris* (20.3%) and *Tetradesmus dimorphus* (21.17%), had similar palmitic acid content, but species such as *Halamphora subtropica* (37.9%) and *Coelastrum microporum* (25.66%) exhibited higher concentrations (Table S1). Additionally, *D. salina* DSTA20 contained significantly more palmitic acid than other species, such as *Chaetoceros muelleri* (11.3%) and *Nannochloropsis oculata* (18.9%). Even when compared to second-generation oil sources, such as jatropha (13.4%) and karanja (7.4%), *D. salina* DSTA20 offers higher concentrations, reinforcing its potential for industrial applications, especially in biofuels. Although palm oil contains the highest concentration of palmitic acid (47.9%), *D. salina* DSTA20 is a more sustainable alternative because of the reduced land and water requirements for microalgal cultivation [74]. Thus, *D. salina* DSTA20 is a viable candidate for biodiesel production and other industrial applications.

To evaluate the biodiesel quality of *D. salina* DSTA20 compared with that of terrestrial plants and other microalgae, we analyzed its biodiesel properties based on FAME profiles. Key biodiesel parameters, such as IV, CN, and OS, are critical for assessing diesel engine performance because they influence the combustion quality, storage stability, and cold flow characteristics, making them essential for the practical application of biodiesel [33,77,78]. Specific values for high-quality biodiesel are defined by the European standard EN14214 and American standard ASTM D6751-02. *D. salina* DSTA20 meets several of these standards. The OS value of 8.74 exceeds both the EN14214 requirement of ≥6 and the ASTM D6751-02 requirement of ≥3, indicating strong OS, crucial for long-term storage and performance. Additionally, the CFPP of −9.03 °C is within the acceptable range of ≤−20 to 5 °C under EN14214, ensuring good cold flow properties for biodiesel in colder environments. However, some biodiesel properties of *D. salina* DSTA20 did not meet the required standards. The IV of 169.05 exceeds the EN14214 limit of 120, indicating a higher degree of unsaturation, which could negatively impact oxidative stability and cold flow properties. Additionally, the CN of 35.04 is below the EN14214 and ASTM D6751-02 minimum requirements of 51 and 47, respectively, suggesting reduced ignition quality. In comparison to terrestrial crops, such as jatropha, karanja, palm, mahua, and rapeseed, *D. salina* DSTA20 also showed a higher IV and lower CN. To address these shortcomings, *D. salina* DSTA20 biodiesel can be blended with other biodiesel sources with lower IV and higher CN values to achieve a balanced fuel profile suitable for diesel engines [79,80]. Targeted adjustments to these parameters could improve the quality and yield of biodiesel derived from *D. salina* DSTA20. Compared to other microalgal strains, such as *Chlamydomonas hedleyi* MM0020, *Chlorella salina* MM0063, and *D. salina* LIMS-PS-1511, *D. salina* DSTA20 exhibited a relatively higher IV, similar to other high-unsaturation strains, such as *Mychonastes homosphaera* UTEX 2341. However, the CN of *D. salina* DSTA20 remained lower than those of most of these microalgae, emphasizing the need for further optimization or blending strategies to achieve a balanced fuel profile for use in diesel engines.

### 4.5. Carotenoid Composition

Carotenoid analysis revealed that *D. salina* DSTA20 produces significant amounts of β-carotene, lutein, and zeaxanthin, with β-carotene being the dominant carotenoid. The optimal salinity for carotenoid production was found to be 0.25 M, at which β-carotene, lutein, and zeaxanthin concentrations peaked at 2.47, 1.39, and 0.69 mg g^−1^ DW, respectively. This aligns with findings from earlier studies indicating that salinity levels play a crucial role in carotenoid accumulation in *D*. *salina* [81,82,83]. However, the lack of consistency across different strains, considering some studies have reported no significant changes in carotenoid levels under high salinity [84], suggests that strain-specific responses and local environmental adaptations may influence carotenoid production. Further research on the genetic and environmental factors governing these processes in *D. salina* is necessary to better understand these discrepancies.

A comparison with other species reveals that green microalgae, such as *Haematococcus lacustris*, accumulate specific carotenoids to adapt to extreme environments. *H. lacustris* stores astaxanthin to withstand high light intensity and nutrient-deficient conditions [85]. Additionally, the *Bracteacoccus* species and *Botryococcus braunii* utilize a multi-pigment strategy, accumulating carotenoids, such as adonirubin, echinenone, and botryoxanthins, to manage light and oxidative stress [85]. Notably, some species, such as *Chlorosarcinopsis dissociata*, accumulate both astaxanthin and canthaxanthin, a unique trait not observed in *D. salina*. These comparisons highlight the distinctive adaptation of *D. salina* to selectively accumulate β-carotene as a mechanism for enhancing resilience in high-salinity environments, contrasting with the mixed pigment strategies seen in species adapted to high-light and low-nutrient conditions [85].

Carotenoids, especially β-carotene, are valued for their pro-vitamin A activity and antioxidant properties, which help reduce oxidative stress and cancer risk [86,87]. This demand spans the food, pharmaceutical, and cosmetic industries [88]. Although natural sources, such as vegetable and fruit waste, have been explored [89,90], halotolerant microalgae such as *D*. *salina* are the most promising because of their high pigment accumulation under specific conditions [91,92]. The β-carotene content of *D*. *salina* DSTA20 at 2.47 mg g^−1^ DW is lower than the levels reported in other *D*. *salina* strains. In particular, the reference strain documented by Ben-Amotz [92] achieved, β-carotene levels of up to 100 mg g^−1^ DW.

Future studies should aim at enhancing β-carotene productivity in *D*. *salina* DSTA20 by testing stress conditions, such as varying salinity, PFD, and nutrient availability, while exploring genetic or metabolic engineering approaches to further optimize carotenoid biosynthesis. Despite the lower β-carotene yield compared with that in the strain used by Ben-Amotz, *D*. *salina* DSTA20 demonstrates higher production than other species, such as *Graesiella emersonii* GEGS21 (0.84 mg g^−1^ DW [13]) and *Phaeodactylum tricornutum* CCMP1327 (1.6 mg g^−1^ DW, [93]), indicating its potential for industrial use. *D*. *salina* DSTA20 may serve as a valuable bioresource for carotenoid production, especially under conditions optimized for β-carotene synthesis.

### 4.6. Monosaccharide Composition

The glucose content of *D*. *salina* DSTA20 was compared with that of other microalgal strains and terrestrial plants. *D*. *salina* DSTA20 exhibited a glucose concentration of 195.5 mg g^−1^ DW, which is notably high among microalgal species. For instance, the glucose content of *Chlorella salina* is 124.1 mg g^−1^ DW, and *Picochlorum atomus* contains 55.2 mg g^−1^ DW. Compared with terrestrial plants, the glucose content of *D*. *salina* DSTA20 exceeds that of most plants, except for cabbage (258.5 mg g^−1^ DW). For example, grape contains 108.2 mg g^−1^ DW of glucose, and sweet potato contains only 22.8 mg g^−1^ DW. Glucose plays an important role in bioethanol production [94], and in third-generation bioethanol production, microalgal biomass can serve as a source of various fermentable carbohydrates, including glucose [95]. Unlike the first-generation bioethanol, which relies on food crops, such as corn and sugarcane, and second-generation bioethanol that uses lignocellulosic materials and requires complex pretreatment, microalgae offer distinct advantages [96]. Microalgae such as *D*. *salina* grow faster, accumulate carbohydrates more efficiently, and require less land, making them a more sustainable source for bioethanol production [97,98]. Additionally, they absorb large amounts of carbon dioxide, further enhancing their environmental benefits [99]. In addition to bioethanol, the glucose from *D*. *salina* has significant applications in the cosmetics industry, where it is used in products such as baby care, oral hygiene, and spray formulations [100]. Moreover, glucose plays a crucial role in the production of polylactic acid, a biodegradable plastic that has versatile industrial applications and supports the development of environmentally friendly materials [101]. In conclusion, the high glucose content of *D*. *salina* DSTA20 makes it a promising resource for diverse industries, including bioethanol, cosmetics, food, and chemical products. Its high carbohydrate accumulation, combined with its rapid growth and environmental benefits, suggests that *D*. *salina* DSTA20 has significant potential for industrial applications across multiple sectors.

*D. salina* has gained widespread recognition and approval in various industries, particularly in cosmetics and nutrition [17]. In the cosmetics sector, it is utilized for its bioactive properties, such as the stimulation of cell proliferation, as demonstrated by products such as Pepta-Ctive and blue retinol [17]. Officially approved as a food additive in European countries including France, Italy, and Belgium [102], *D. salina* has also earned the Generally Recognized as Safe status from the U.S. Food and Drug Administration, facilitating its use in both human and animal nutrition [103]. Furthermore, it is recognized as a safe food ingredient in Canada and China, where it is commercially available [104]. Given its existing approval and growing demand, *D. salina* DSTA20 has significant potential as an industrial feedstock for various biotechnological applications and its regulatory approval is expected to expand further into new markets.

## 5. Conclusions

In this study, we examined the taxonomic, physiological, and biochemical characteristics of the halophilic microalga *D*. *salina* DSTA20. Accurate taxonomic identification, confirmed through morphological and molecular analyses, is essential to understand its ecological role and maximize its industrial potential. Notably, *D*. *salina* DSTA20 demonstrated better growth and higher carotenoid production at lower salinities than typical halophilic organisms. The strain also produces a significant proportion of industrially valuable fatty acids under these conditions, which makes it well suited for biofuel production, health supplements, and other commercial applications.

By registering *D*. *salina* DSTA20 with the National Marine Biodiversity Institute of Marine BioBank, we have established a critical resource for future research and industrial applications. These findings offer valuable insights into the adaptability and metabolic flexibility of *D*. *salina* DSTA20, making it a promising resource for sustainable biotechnology. This study lays the groundwork for further research to optimize the cultivation conditions and explore the genetic traits of this strain to enhance its commercial viability in various industries.

## Figures and Tables

**Table 1:** Strain, location of collection (LC), water temperature (T, °C), salinity (S, PSU), and GenBank accession numbers (GBAN) for marker genes of *Dunaliella salina* DSTA20 isolated from the Naeri Mandae Solhyanggi Salt Pond in Taean-gun, Chungcheongnam-do, Republic of Korea.

Species	Strain	LC	Date	T (°C)	S (PSU)	Marker Gene	Amplicon Length (bp)	GBAN
*D. salina*	DSTA20	Mandae Solhyanggi-gil Salt pond	July 2020	27.2	>100	SSU	1460	PP973483
						ITS	602	PP974559
						LSU	746	PP973494
						*rbc*L	345	PP975467

SSU (small subunit ribosomal DNA), ITS (internal transcribed spacer), LSU (large subunit ribosomal DNA), *rbc*L (ribulose-1,5-bisphosphate carboxylase/oxygenase large subunit).

**Table 2:** Primers used to amplify the small (SSU), large subunit (LSU), and internal transcribed spacer 1 (ITS1)-5.8S-internal transcribed spacer 2 (ITS2) regions of rDNA and the *rbc*L genes of *Dunaliella salina* DSTA20.

Primer Name	Primer Region	Sequence (5′-3′)	References
EukA	Forward, SSU	AACCTGGTTGATCCTGCCAG	[24]
G18R	Reverse, SSU	GCATCACAGACCTGTTATTG	[25]
570F	Forward, SSU	GTAATTCCAGCTCCAATAGC	[26]
EukB	Reverse, SSU	TGATCCTTCTGCAGGTTCACCTAC	[24]
ITSF2	Forward, ITS	ACCCGCTGAATTTAAGCATA	[25]
ITSFR2	Reverse, ITS	ACGAACGATTTGCACGTCAG	[25]
D1R	Forward, LSU	ACCCGCTGAATTTAAGCATA	[27]
LSUB	Reverse, LSU	ACGAACGATTTGCACGTCAG	[25]
*rbc*L-192	Forward, *rbc*L	GGTACTTGGACAACWGTWTGGAC	[28]
*rbc*L-657	Reverse, *rbc*L	GAAACGGTCTCKCCARCGCAT	[28]

**Table 3:** Comparison of morphological and ultrastructural characteristics of *Dunaliella salina* strains.

Traits	*Dunaliella salina* Strains			
Cell shape	Spherical or oval	Elongated ellipsoidal or cylindrical	Spherical or oval	Oval
Cell length (μm)	9.3–14 (11.3)	16–24, up to 28 for aged zoospores	5–29 (10.9–16.9)	10.2–15.4 (14.1)
Cell width (μm)	7.8–10 (8.1)	ND	3.8–20.3 (7.9–13.2)	9.8–15 (11.1)
Flagella	Two flagella, approximately equal to or longer than cell length in some cells	Two flagella, longer than the total body length	Two flagella, approximately equal to cell length	Two flagella
Chloroplast	Cup-shaped	Bell-shaped	Cup-shaped	Cup-shaped
Pyrenoid	Present, surrounded by the starch grains	Present, surrounded by the starch grains	Present, surrounded by the starch grains	Present, surrounded by the starch grains
Eyespots	Present	Present	Present	Present
Aplanospores	Aplanospores present, spherical, 12–19 (14.6) μm in diameter, with a thick, rugose wall	ND	Aplanospores present, spherical, 12–20 μm in diameter, with a thick, rugose wall	ND
Reference	This study	[35]	[10]	[36,37]

ND: Information not available.

**Table 4:** Comparison of small subunit (SSU) rDNA and *rbc*L sequences of *Dunaliella salina* DSTA20 isolated from Mandae Solhyanggi-gil Salt Pond, Republic of Korea, with those of other strains.

Marker Gene	Collection Location	Strain Habitat (Isolation Source)	Strain Name	GenBank Accession No.	*Dunaliella salina* DSTA20 *
SSU	Australia	Hypersaline	CCAP 19/18	EF473745	0 (0)
	Israel	Hypersaline	SAG 42.88	EF473740	0 (0)
	Israel	Brackish	CCAP 19/12	KJ756842	0 (0)
	Republic of Korea	Hypersaline	KMMCC 1428	JQ315781	0 (0)
	ND	ND	GY-H13	MN832905	0 (0)
	Mexico	Hypersaline	UTEX LB 1644	DQ009765	2 (0.1)
	Thailand	soil	KU07	KF825551	7 (0.5)
*rbc*L	China	ND	OUC66	DQ173086	0 (0)
	China	ND	OUC36	DQ173084	0 (0)
	Inner Mongolia	ND	Inner Mongolia	DQ173087	0 (0)
	China	ND	OUC38	DQ173085	0 (0)
	ND	ND	TS-1	AB127990	0 (0)
	USA	ND	UTEX2538	DQ313194	0 (0)
	Republic of Korea	ND	KMMCC1346	JQ315489	0 (0)

* Numbers indicate the number of base pairs that differ from *D. salina* DSTA20 between strains. Numbers in parentheses indicate dissimilarities (%), including gaps. ND: Information not available.

**Table 5:** Fatty acid profiles of *Dunaliella salina* DSTA20 under 0.5 M salinity conditions.

Component	Content (%)	Note
Myristic acid (C_14_:0)	0.49	
Palmitic acid (C_16:0_)	21.06	SFA (major)
Palmitoleic acid (C_16:1_ ω-7)	1.63	
Hexadecadienoic acid (C_16:2_ ω-6)	1.08	
Hexadecatrienoic acid (C_16:3_ ω-3)	2.75	
Hexadecatetraenoic acid (C_16:4_ ω-3)	13.23	ω-3 PUFA (major)
Stearic acid (C_18:0_)	0.53	
Oleic acid (C_18:1_ ω-9)	2.77	
Linoleic acid (C_18:2_ ω-6)	6.81	ω-6 PUFA (major)
γ-linolenic Acid (C_18:3_ ω-6)	3.84	
α-linolenic acid (C_18:3_ ω-3)	31.55	ω-3 PUFA (major)
Total saturated fatty acids	22.08	
Total monounsaturated fatty acids	4.40	
Total polyunsaturated fatty acids	59.26	

SFA, saturated fatty acids; PUFA, polyunsaturated fatty acids.

**Table 6:** Biodiesel properties calculated from the fatty acid methyl ester compositions of the isolated algal strain and other crops and the biodiesel standards EN 14214 [38] and ASTM D6751-02 [39].

Source	SV (mg KOH/g)	IV (g I_2_/100 g)	DU	MUFA (%)	PUFA (%)	LCSF	CFPP (°C)	CN	OS (h)
Jatropha	190.98	105.42	122.1	37.3	42.4	4.54	−2.21	51.16	5.37
Karanja	184.05	94.22	105.2	65.6	19.8	2.64	−8.18	54.76	8.55
Mahua	191.58	67.72	78.62	39.1	19.76	11.65	20.12	59.55	8.56
Palm	194.82	48.05	55.7	37.04	9.33	6.91	5.22	63.5	15.23
Rapeseed	188.61	115.07	125.46	64.4	30.53	0.77	−14.05	49.35	6.45
*Dunaliella salina* DSTA20	171.39	169.05	122.92	4.4	59.26	2.37	−9.03	35.04	8.7
*Dunaliella salina* LIMS-PS-1511	121.29	95.41	78.3	3.7	37.3	2.73	−7.9	69.83	5.75
*Asterarcys quadricellulare* AQYS21	205.11	171.65	134.04	13.3	60.37	2.99	−7.08	34.29	4.54
*Chlamydomonas hedleyi* MM0020	95.6	62.09	55.0	2.6	26.2	2.43	−8.84	89.42	7.09
*Chlorella salina* MM0063	132.19	100.26	85.0	2.6	41.2	2.75	−7.84	65.03	5.45
*Coelastrum microporum* IBL-C119	181.83	82.61	84.64	45.24	19.7	4.02	−3.84	57.73	8.58
*Graesiella emersonii* GEGS21	204.86	131.06	121.3	22.5	49.4	3.05	−6.89	43.45	4.98
*Haematococcus lacustris*	162.7	98.86	95.81	20.13	37.84	3.82	−4.46	57.6	5.75
*Microglena monadina* NFW3	188.54	166.15	138.24	3.28	67.48	2.71	−7.97	37.86	4.34
*Mychonastes homosphaera* UTEX 2341	142.74	162.79	98.9	23.9	37.5	1.45	−11.92	47.91	23.28
*Jaagichlorella luteoviridis* MM0014	157.6	109.69	110.7	7.1	51.8	2.77	−7.77	56.25	4.87
*Tetradesmus obliquus* MM0026	138.92	98.63	85.4	18.4	33.5	2.45	−8.78	63.4	6.11
EN14214	-	≤120	-	-	-	-	≤−20~5	≥51	≥6
ASTM D6751-02	-	-	-	-	-	-	-	≥47	≥3

-, Indicates data not available.

**Table 7:** Composition of photosynthetic carotenoids in the isolated algal strain *Dunaliella salina* DSTA20 under different salinity conditions.

Carotenoids	Salinity (M)	Retention Time (min)	Peak Area (Arbitrary Units)	Amount (mg g^−1^)
β-carotene	0.1	17.038	110.3	0.98
	0.25	17.048	205.1	2.47
	0.5	17.083	410.8	1.27
	1	17.082	408.1	1.26
	1.5	17.108	16	0.12
	2	17.146	159.8	0.54
Lutein	0.1	7.576	51.4	0.73
	0.25	7.58	118.5	1.39
	0.5	7.596	306.9	0.61
	1	7.595	661.6	1.06
	1.5	7.603	31.7	0.27
	2	7.631	185.9	0.46
Zeaxanthin	0.1	-	-	-
	0.25	8.435	14	0.69
	0.5	8.45	52	0.65
	1	8.447	30.8	0.39
	1.5	-	-	-
	2	8.486	30.8	0.39

-, indicates data not available or below the detection limits.

**Table 8:** Comparative monosaccharide composition (mg g^−1^ DW, dry weight) of *Dunaliella salina* DSTA20, other microalgae, and terrestrial plants.

Species	Strain	Monosaccharides (mg g^−1^ DW)				References
		Arabinose	Fructose	Galactose	Glucose	
*Dunaliella salina*	DSTA20	-	13.2	15.7	195.5	This study
*D. salina*	LIMS-PS-1511	-	-	26.9	107.4	[40]
*D. tertiolecta*	CS-175	0.65	-	1.1	85.3	[41]
*Chlorella salina*	MM0063	27.8	19.0	75.1	124.1	[42]
*Picochlorum atomus*	CS-183	0.16	-	10.6	55.2	[41]
Terrestrial plants						
Sweet potato	-	-	25.3	-	22.8	[43]
Cabbage	-	-	261.8	-	258.5	[43]
Grape	-	-	126.4	-	108.2	[43]

-, indicates data not available or below the detection limits.

## Data Availability

The original data presented in this study are openly available from the National Marine Biodiversity Institute of Korea and the Korean Collection for Type Cultures at MABIK LP00000338 and KCTC 15718BP.

## Supplementary Materials

The following supporting information can be downloaded at: https://www.mdpi.com/article/10.3390/microorganisms12122467/s1, Figure S1: Phylogenetic tree (NJ tree) based on internal transcribed spacer (ITS) sequence analysis of *Dunaliella* strains, with our strain, *Dunaliella salina* DSTA20 (PP974559), included within the *Dunaliella salina* clade 2. The tree was constructed using the neighbor-joining (NJ) method with 1000 bootstrap replicates to ensure statistical reliability. Comparative strain and species data were obtained from NCBI, and the analysis was conducted using Geneious Prime v.2024.0.7; Table S1: Comparison of major fatty acid percentages between *Dunaliella salina* DSTA20 and other strains, microalgae, and selected second-generation oil sources. BAC, Bacillariophyta (diatoms); CHL, Chlorophyta; HAP, Haptophyta; OCH, Ochrophyta. References [40,75,84,105,106,107,108,109,110,111,112,113,114,115,116,117,118,119,120,121,122,123,124,125,126,127] are cited in the Supplementary Materials.
