# Supplementary material for: Taxonomical, Physiological, and Biochemical Characteristics of Dunaliella salina DSTA20 from Hypersaline Environments of Taean Salt Pond, Republic of Korea
Source: Microorganisms. 2024 Nov 30;12(12):2467. doi: 10.3390/microorganisms12122467 (PMC11676334; doi:10.3390/microorganisms12122467)
Supplement: Supplementary file 1 [file microorganisms-12-02467-s001.zip › microorganisms-3303672-supplementary.pdf]

microorganisms

Table S1. Comparison of major fatty acid percentages between *Dunaliella salina* DSTA20 and other strains, microalgae, and selected second-generation oil sources. BAC, Bacillariophyta (diatoms); CHL, Chlorophyta; HAP, Haptophyta; OCH, Ochrophyta.

Source	Group	Strain	Major individual fatty acids of *D. salina* DSTA20 as percentages of total fatty acids for comparative analysis				References
			Palmitic	Hexadecatetraenoic	Linoleic	α-Linolenic	
			$C_{16:0}$	$C_{16:4}$ ω3	$C_{18:2}$ ω6	$C_{18:3}$ ω3	
Dunaliella salina	CHL	DSTA 20	21.06	13.23	6.81	31.55	This study
D. salina	CHL	CCAP 19/12	12.16	-	-	-	[1]
D. salina	CHL	Y6	19.73	-	3.47	39.51	[2]
D. salina	CHL	ITC5.103	23.43	-	9.68	35.95	[3]
D. salina	CHL	CCAP 19/18	16.33	-	-	-	[4]
D. salina	CHL	LIMS-PS-1511	19.3	-	5.6	31.7	[5]
Asterarcys quadricellulare	CHL	-	17.17	10.06	3.68	24.32	[6]
Botryococcus braunii	CHL	IBL-C117	7.17	-	5.16	5.34	[7]
Chaetoceros calcitrans	BAC	CCMP 1315	7.9	-	0.2	0.4	[8]
C. muelleri	BAC	CCAP 1010/3	11.3	-	0.4	0.1	[8]
Chlamydomonas hedleyi	CHL	MM0020	18.3	-	9.8	16.4	[9]
Mychonastes homosphaera	CHL	UTEX 2341	11.9	-	-	-	[10]
Chlorella vulgaris	CHL	CCAP 211/8K	20.3	-	-	-	[11]

Chlorella vulgaris	CHL	KNUA007	22.35	-	17.97	36.14	[5]
Chlorococcum humicola	CHL	UP08	20.3	-	-	-	[12]
Coelastrum microporum	CHL	IBL-C119	25.66	-	8.58	-	[7]
Haematococcus lacustris	CHL	-	22.49	-	20.23	16.18	[13]
Halamphora subtropica	BAC	UMACC 370	37.9	-	1.5	-	[14]
Jaagichlorella luteoviridis	CHL	MM0014	20.7	-	35.6	16.2	[15]
Hindakia sp.	CHL	PKU AC 169	17.17	-	11.28	20.08	[16]
Monoraphidium sp.	CHL	-	19.01	-	22.51	17.53	[17]
Monoraphidium sp.	CHL	HDMA-20	23.1	16.4	-	35.4	[18]
Nannochloropsis oculata	OCH	CCAP 849/1	18.9	-	2.1	1.1	[8]
Odontella aurita	BAC	OAOSH22	15.96	-	0.3	-	[25]
Oocystis submarina	CHL	-	17.17	-	16.98	28.00	[17]
Pinnularia insolita	BAC	VP280	23.4	-	0.2	-	[19]
P. microgibba	BAC	VP289	20.1	-	5.5	-	[19]
Platessa lutheri	BAC	ATCC 50092	10.7	-	-	-	[10]
Tetradesmus dimorphus	CHL	RW40	21.17	-	-	-	[12]
Tetradesmus obliquus	CHL	UP09	18.21	-	-	-	[12]

Scenedesmus sp.	CHL	NC1	24.54	-	10.42	-	[20]
Selenastrum sp.	CHL	RW09	18.92	-	-	-	[12]
Tisochrysis lutea	HAP	CCAP 927/14	4.6	-	3.5	7.3	[8]
Second-generation oil sources							
Jatropha	-	-	13.4	-	42.1	0.2	[21]
Karanja	-	-	7.4	-	15.4	-	[22]
Mahua	-	-	21.36	-	19.47	-	[23]
Palm	-	-	47.9	-	9.07	-	[24]
Rapeseed			3.49		22.3		[26]

-, indicates data not available.

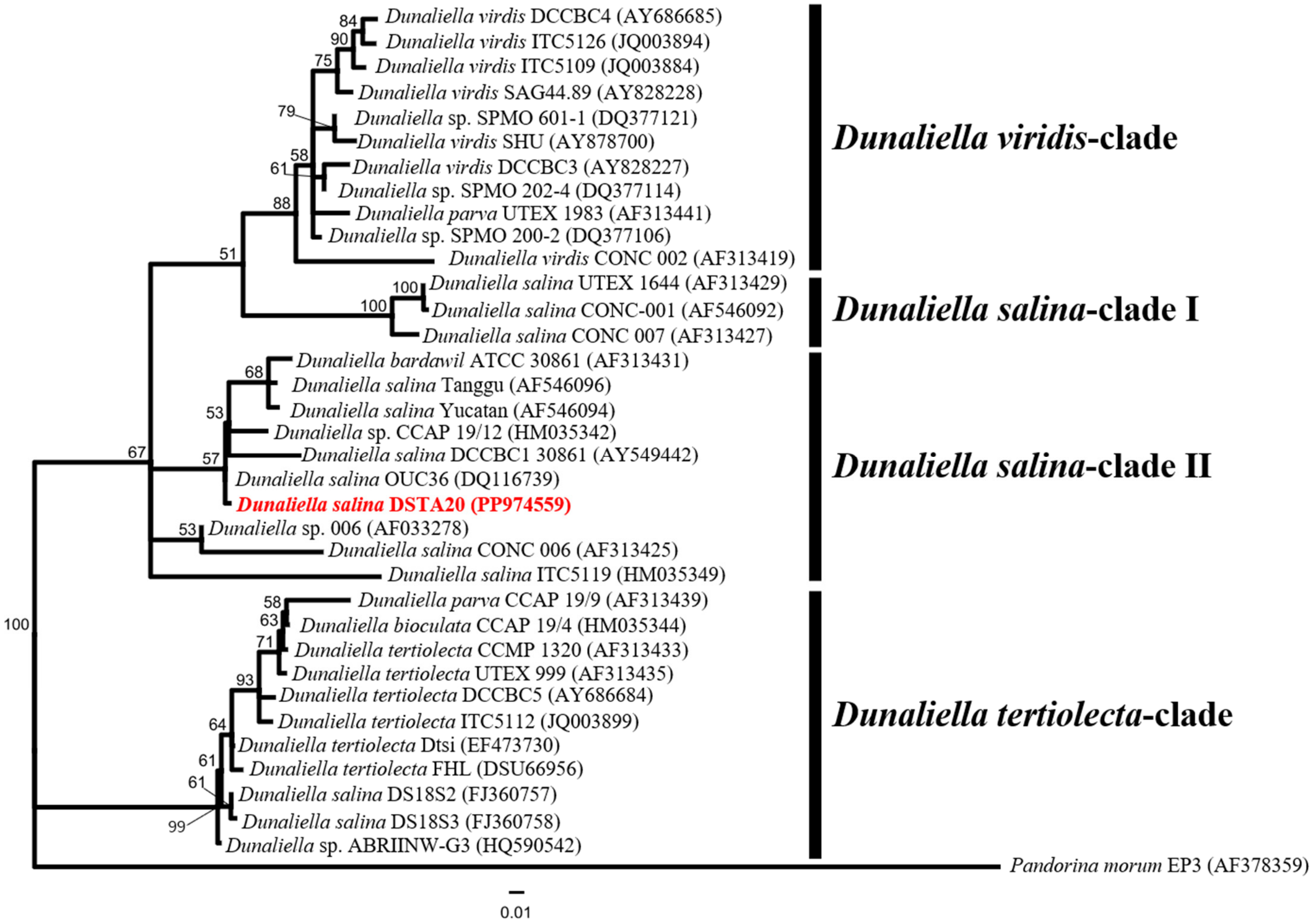

Figure S1. Phylogenetic tree (NJ tree) based on ITS sequence analysis of *Dunaliella* strains, with our strain, *D. salina* DSTA20 (PP974559), included within the *Dunaliella salina* clade 2. The tree was constructed using the Neighbor-Joining (NJ) method with 1,000 bootstrap replicates to ensure statistical reliability. Comparative strain and species data were obtained from NCBI, and the analysis was conducted using Geneious Prime v.2024.0.7.

References

1. Almutairi, A.W. Effects of nitrogen and phosphorus limitations on fatty acid methyl esters and fuel properties of *Dunaliella salina*. *Environ. Sci. Pollut. Res. Int.* **2020**, *27*, 32296–32303, https://doi.org/10.1007/s11356-020-08531-8.
2. Wu, M.; Zhu, R.; Lu, J.; Lei, A.; Zhu, H.; Hu, Z.; Wang, J. Effects of different abiotic stresses on carotenoid and fatty acid metabolism in the green microalga *Dunaliella salina* Y6. *Ann. Microbiol.* **2020**, *70*, 48, https://doi.org/10.1186/s13213-020-01588-3.
3. Mendoza Guzmán, H.; de la Jara Valido, A.; Freijanes Presmanes, K.; Carmona Duarte, L. Quick estimation of intraspecific variation of fatty acid composition in *Dunaliella salina* using flow cytometry and Nile Red. *J. Appl. Phycol.* **2012**, *24*, 1237–1243, https://doi.org/ 10.1007/s10811-011-9768-y.
4. Talebi, A.F.; Tohidfar, M.; Bagheri, A.; Lyon, S.R.; Salehi-Ashtiani, K.; Tabatabaei, M. Manipulation of carbon flux into fatty acid biosynthesis pathway in *Dunaliella salina* using *AccD* and *ME* genes to enhance lipid content and to improve produced biodiesel quality. *Biofuel Res. J.* **2014**, *1*, 91–97.
5. Jo, S.W.; Do, J.M.; Kang, N.S.; Park, J.M.; Lee, J.H.; Kim, H.S.; Hong, J.W.; Yoon, H.S. Isolation, identification, and biochemical characteristics of a cold-tolerant *Chlorella vulgaris* KNUA007 isolated from King George Island, Antarctica. *J. Mar. Sci. Eng.* **2020**, *8*, 935. https://doi.org/10.3390/jmse8110935.
6. Mohmmed, S. Screening of four green microalgae potentially used as feedstock for biodiesel and nutraceuticals. *J. Appl. Phycol.* **2022**, *34*, 1565–1581.
7. Nascimento, I.A.; Marques, S.S.I.; Cabanelas, I.T.D.; Pereira, S.A.; Druzian, J.I.; De Souza, C.O.; De Vich, D.V.; De Carvalho, G.C.; De Nascimento, M.A. Screening microalgae strains for biodiesel production: Lipid productivity and estimation of fuel quality based on fatty acids profiles as selective criteria. *Bioenergy Res.* **2013**, *6*, 1–13, https://doi.org/10.1007/s12155-012-9222-2.
8. Reitan, K.I.; Øie, G.; Jørgensen, H.; Wang, X. Chemical composition of selected marine microalgae, with emphasis on lipid and carbohydrate production for potential use as feed resources. *J. Appl. Phycol.* **2021**, *33*, 3831–3842, https://doi.org/10.1007/s10811-021-02586-x.
9. Jo, S.W.; Kang, N.S.; Lee, J.A.; Kim, E.S.; Kim, K.M.; et al. Characterization of MABIK microalgae with biotechnological potentials. *J. Mar. Sci. Eng.* **2020**, *12*, 40–49.
10. Vazhappilly, R.; Chen, F. Eicosapentaenoic acid and docosahexaenoic acid production potential of microalgae and their heterotrophic growth. *J. Amer. Oil Chem. Soc.* **1998**, *75*, 393–397, https://doi.org/ 10.1007/s11746-998-0057-0.
11. Afi, L.; Metzger, P.; Largeau, C.; Connan, J.; Berkaloff, C.; Rousseau, B. Bacterial degradation of green microalgae: Incubation of *Chlorella emersonii* and *Chlorella vulgaris* with *Pseudomonas oleovorans* and *Flavobacterium aquatile*. *Org. Geochem.* **1996**, *25*, 117–130, https://doi.org/10.1016/S0146-6380(96)00113-1.
12. Kabir, F.; Gulfraz, M.; Raja, G.K.; Inam-ul-Haq, M.; Awais, M.; Mustafa, M.S.; Khan, S.U.; Tlili, I.; Shadloo, M.S. Screening of native hyper-lipid producing microalgae strains for biomass and lipid production Screening of native hyper-lipid producing microalgae strains for biomass and lipid production. *Renew. Energy* **2020**, *160*, 1295–1307, https://doi.org/10.1016/j.renene.2020.07.004.
13. Damiani, M.C.; Popovich, C.A.; Constenla, D.; Leonardi, P.I. Lipid analysis in *Haematococcus pluvialis* to assess its potential use as a biodiesel feedstock. *Bioresour. Technol.* **2010**, *101*, 3801–3807, https://doi.org/10.1016/j.biortech.2009.12.136.
14. Vello, V.; Phang, S.M.; Poong, S.W.; Lim, Y.K.; Ng, F.L.; Shanmugam, J.; Gopal, M. New report of *Halamphora subtropica* (Bacillariophyta) from the Strait of Malacca and its growth and biochemical characterisation under nutrient deprivation. *Reg. Stud. Mar. Sci.* **2023**, *62*, 102947, https://doi.org/10.1016/j.rsma.2023.102947.
15. Kim, K.M.; Kang, N.S.; Jang, H.S.; Park, J.S.; Jeon, B.H.; Hong, J.W. Characterization of *Heterochlorella luteoviridis* (Trebouxiaceae, Trebouxiophyceae) isolated from the Port of Jeongja in Ulsan, Korea. *J. Mar. Biosci. Biotechnol.* **2017**, *9*, 22–29, https://doi.org/10.15433/ksmb.2017.9.2.022.
16. Daroch, M.; Shao, C.; Liu, Y.; Geng, S.; Cheng, J.J. Induction of lipids and resultant FAME profiles of microalgae from coastal waters of Pearl River Delta. *Bioresour. Technol.* **2013**, *146*, 192–199, https://doi.org/10.1016/j.biortech.2013.07.048.
17. Hawrot-Paw, M.; Ratomski, P.; Koniuszy, A.; Golimowski, W.; Teleszko, M.; Grygier, A. Fatty acid profile of microalgal oils as a criterion for selection of the best feedstock for biodiesel production. *Energies* **2021**, *14*, 7334, https://doi.org/10.3390/en14217334.
18. Lin, Y.; Ge, J.; Zhang, Y.; Ling, H.; Yan, X.; Ping, W. *Monoraphidium sp.* HDMA-20 is a new potential source of α-linolenic acid and eicosatetraenoic acid. *Lipids Health Dis.* **2019**, *18*, 56, https://doi.org/10.1186/s12944-019-0996-5.
19. Kezlya, E.; Maltsev, Y.; Genkal, S.; Krivova, Z.; Kulikovskiy, M. Phylogeny and fatty acid profiles of new *Pinnularia* (Bacillariophyta) species from soils of Vietnam. *Cells* **2022**, *11*, 2446, https://doi.org/10.3390/cells11152446.

20. Kumar, N.; Banerjee, C.; Jagadevan, S. Identification, characterization, and lipid profiling of microalgae *Scenedesmus sp.* NC1, isolated from coal mine effluent with potential for biofuel production. *Biotechnol. Rep.* **2021**, *30*, e00621, https://doi.org/10.1016/j.btre.2021.e00621.
21. Becker, K.; Makkar, H.P.S. *Jatropha curcas*: A potential source for tomorrow's oil and biodiesel. *Lipid Technol.* **2008**, *20*, 104–107, https://doi.org/10.1002/lite.200800023.
22. Goembira, F.; Saka, S. Advanced supercritical methyl acetate method for biodiesel production from *Pongamia pinnata* oil. *Renew. Energy* **2015**, *83*, 1245–1249, https://doi.org/10.1016/j.renene.2015.06.022.
23. Saravanan, N.; Nagarajan, G.; Puhan, S. Experimental investigation on a DI diesel engine fuelled with *Madhuca indica* ester and diesel blend. *Biomass Bioenergy* **2010**, *34*, 838–843, https://doi.org/10.1016/j.biombioe.2010.01.028.
24. Crabbe, E.; Nolasco-Hipolito, C.; Kobayashi, G.; Sonomoto, K.; Ishizaki, A. Biodiesel production from crude palm oil and evaluation of butanol extraction and fuel properties. *Process Biochem.* **2001**, *37*, 65–71, https://doi.org/10.1016/S0032-9592(01)00178-9.
25. An, S.M.; Cho, K.; Kim, E.S.; Ki, H.; Choi, G.; Kang, N.S. Description and characterization of the *Odontella aurita* OAOSH22, a marine diatom rich in eicosapentaenoic acid and fucoxanthin, isolated from Osan Harbor, Korea. *Mar. Drugs* **2023**, *21*, 563.
26. Ramadhas, A.S.; Muraleedharan, C.; Jayaraj, S. Performance and emission evaluation of a diesel engine fueled with methyl esters of rubber seed oil. *Renew. Energy* **2005**, *30*, 1789–1800.
